# RARE PRESENTATION OF KYRLE'S DISEASE IN SIBLINGS

**DOI:** 10.4103/0019-5154.41654

**Published:** 2008

**Authors:** Seethalakshmi Viswanathan, Swati D Narurkar, Aruna Rajpal, N G Nagpur, S S Avasare

**Affiliations:** *From Department of Pathology, KJ Somaiya Medical College, Sion, Ayurvihar, Mumbai, Maharashtra, India*; 1 *From Department of Dermatology, KJ Somaiya Medical College, Sion, Ayurvihar, Mumbai, Maharashtra, India*

**Keywords:** *Familial*, *Kyrle's*, *metabolic*, *perforating dermatoses*, *primary*

## Abstract

**Background:**

Kyrle's disease is a rare variant of primary perforating dermatosis. Its occurrence in a familial setting, especially in children, is extremely uncommon. Similar appearing skin lesions have been described in adults, secondary to metabolic disorders, infective agents as well as exposure to chemicals. We present a rare case of this genodermatosis in two siblings.

**Materials and Methods:**

Two siblings of a non-consanguineous marriage came with generalized discrete papular lesions with a central keratotic plug. All biochemical and serological investigations were within normal limits. Serial sections of the biopsy revealed typical epidermal invaginations filled with parakeratotic debris and perforation into the dermis with accompanying granulomatous reaction.

**Results and Conclusions:**

A careful history, detailed routine investigations and serial sections of the skin biopsy are required to demonstrate the typical morphology and stages of evolution of Kyrle's disease. This helps to differentiate the rare primary Kyrle's disease from other primary and secondary keratotic lesions. Due to the familial occurrence, screening of relatives of an index case is recommended.

## Introduction

Perforating dermatosis represents a heterogeneous group of disorders characterized by transepithelial elimination of dermal structures.^1^,^2^ Primary perforating disorders include Kyrle's disease (hyperkeratosis follicularis et parafollicularis in cutem penetrans), elastosis perforans serpiginosa, perforating folliculitis and reactive perforating collagenosis.^1^,^3^ They need to be distinguished from the secondary perforating dermatosis due to metabolic conditions, such as uraemia, especially in patients on dialysis, diabetes, hepatic failure or as a paraneoplastic syndrome in multiple myeloma.^4^–^9^ An infective aetiology^10^ and exposure to chemical agents^11^ have also been reported to be causative factors for Kyrle's disease.

## Case History

**Fig. 1:**
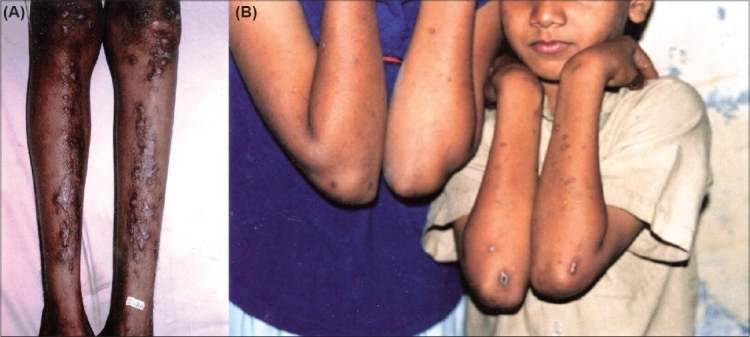
(A) Multiple discrete keratotic papules with peripheral scaling and central umbilication in the lower extremities (male patient). (B) Similar lesions in the dorsum of the hand with a central keratotic plug (female patient)

Two healthy siblings of a non-consanguineous marriage, a 7-year-old male child and his 10-year-old sister presented with multiple spontaneously occurring mildly itchy lesions over the limbs, face and trunk since 2 and 4 years of age, respectively. There was no preceding history of trauma and no signs or symptoms of any systemic disease, metabolic condition or any infection. A possible prior application of any chemical substance was ruled out on repeated interrogation of the obviously anxious mother. On examination, there were generalized multiple discrete keratotic papules with peripheral scaling and central umbilication. The central keratotic plug could be removed and the lesions revealed Koebner's phenomenon (Fig. 1) and healed with hyperpigmentation. They did not involve mucous membranes, palmar or plantar surfaces. All biochemical and serological investigations, including liver and renal function tests, were within normal limits.

**Fig. 2:**
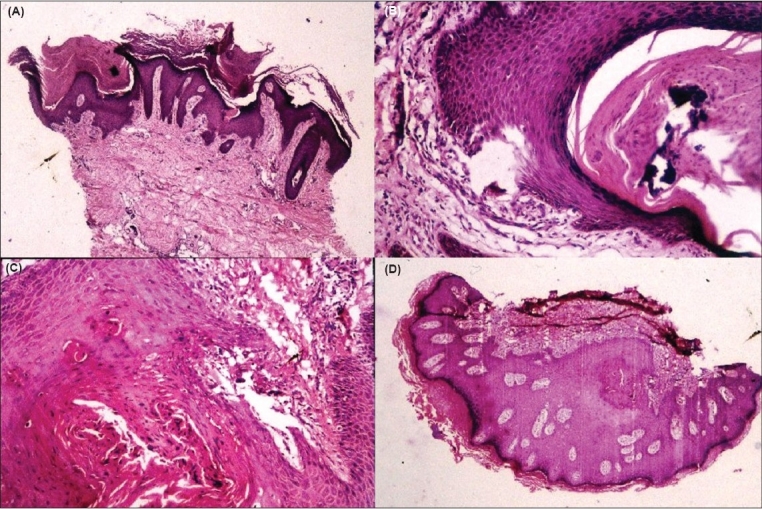
Multiple serial cuts of the biopsy showing various stages of the lesion. (A) Biopsy lined by hyperplastic hyperkeratotic epidermis with multiple epidermal invaginations (H and E, ×40). (B) Invagination containing parakeratotic scale crust with basophilic debris (H and E, ×100). (C) Site of perforation with adjacent dermis showing granulomatous reaction (H and E, ×100). (D) Pseudoepitheliomatous hyperplasia of the surrounding epidermis seen to cover the above process from its base (H and E, ×40)

A skin biopsy was performed from an active lesion. On histopathology, the lesion showed a hyperplastic hyperkeratotic epidermis, a well-developed granular layer and multiple epidermal invaginations containing parakeratotic scale crust with basophilic debris. On serial sectioning, the epidermal invagination showed a focal defect at the dermal interface with an adjacent granulomatous reaction in the dermis. Further serial cuts revealed pseudoepitheliomatous hyperplasia of the surrounding epidermis, which was seen to cover the above process from its base (Fig. 2). No hair structure was seen in our case within any of the invaginations.

## Discussion

Kyrle's disease is a rare cause of primary perforating dermatosis in children. Very rarely a familial predisposition has been reported supporting the view that this represents a genodermatosis with an autosomal recessive trait.^12^–^14^ The disease could also present in adulthood, more commonly in women between 30 and 50 years of age.^12^,^14^

This disorder of keratinization results in the development of dyskeratotic cells at multiple points within epidermal invaginations in the skin.^5^ These cells have a limited capacity for proliferation while retaining an accelerated rate of keratinization and differentiation. Eventually, this results in a depleted cell and a consequent defect in the epidermis.^1^,^3^ The rapid keratinization results in the formation of an overlying parakeratotic column, which then perforates into the dermis, eliciting a granulomatous inflammatory reaction. Subsequent re-epithelization from the adjacent epidermis covers this entire process from the base. The dermal connective tissue, inflammation and the keratotic debris degenerate to form the basophilic debris, which corresponds to the keratotic plug. This is exuded from the invagination seen in the fully evolved form of the lesion.^1^,^4^,^5^,^13^,^15^

All these features could be well demonstrated in the present case due to the diligent study of multiple serial cuts of the biopsy, and hence helped in the confirmation of the diagnosis.

Secondary metabolic causes of perforating dermatosis, a much more common occurrence,^4^–^8^ were ruled out because all investigations pertaining to liver, renal and endocrine functions, including blood sugar levels, were within normal limits. An infective etiology was ruled out in the present case, because the lesions were not concurrent and developed at different points of time during the early childhood of the two siblings and did not regress after a course of antibiotics.

Kyrle's disease needs to be differentiated from other primary perforating dermatosis, such as perforating folliculitis, where the epidermal invagination is in relation to a vellus hair,^16^ which was absent in the present case. Elastosis perforans serpiginosa, associated with Down's syndrome and genetic disorders of connective tissue,^1^,^13^ was ruled out with the absence of thickened elastic fibres around epidermal invaginations, which was also confirmed by the Elastic Von Gieson stain. Reactive perforating collagenosis was not considered due to the lack of degenerated collagen at the base of the perforation.^1^

## Conclusion

To conclude, the presence of an underlying metabolic disorder needs to be ruled out before labelling the case as a primary Kyrle's disease with the help of a detailed history and relevant biochemical and serological investigations. Although familial primary Kyrle's disease is a rarity, first-degree relatives need to be screened in all cases of perforating dermatosis. A proper biopsy processing due to the thick keratotic plug and the importance of serial sections to demonstrate the perforation is stressed.
